# Knowledge, Attitudes, and Practice towards Occupational Health and Safety among Nursing Students in Gaza Strip, Palestine

**DOI:** 10.4314/ejhs.v32i5.16

**Published:** 2022-09

**Authors:** Abdel Fattah A Qaraman, Maher Elbayoumi, Edris Kakemam, Ahmed Hassan Albelbeisi

**Affiliations:** 1 Intermediate Studies College, Israa University, Gaza, Palestine; 2 Energy and Sustainable Environment Center, School of Engineering, Israa University, Gaza, Palestine; 3 Tabriz Health Services Management Research Center, Tabriz University of Medical Sciences, Tabriz, Iran; 4 Medical Services Directorate, Gaza Strip, Palestine

**Keywords:** Knowledge, Attitude, Practice, KAP, Nurses, Gaza Strip

## Abstract

**Background:**

Health and safety in the workplace are critical components in healthcare institutions. Unsafe working conditions are among the causes of poor quality of care and burnout. This study aims to assess the knowledge, attitudes, and practice of occupational health and safety among nursing students at Al-Israa University.

**Methods:**

In this cross-sectional study, a structured online questionnaire was distributed from March to May 2021. Of the 350 eligible students, 219 students answered the questionnaire (Response rate=62.6). Data were analyzed using the statistical software IBM-SPSS version 22. Descriptive statistic, Independentsamples T-Test, and ANOVA tests were used.

**Results:**

The majority of participants were female (81.7%) and studying in a diploma program. 21% of nursing students have experienced a needle stick injury. The mean scores for knowledge, attitudes, and practice were (M±SD:78.2% ±12.9, M±SD:80.6% ±7.1, and M±SD:81.2% ±7.6) respectively. In terms of knowledge, attitudes, and practice the mean scores were statistically significant between nursing students who attended a safety precautions course and those who didn not (P-value <.05). In terms of attitudes, the mean scores were statistically significant between diploma and bachelor students (P-value =.026). In terms of practice, the means scores were statistically significant between males and females (Pvalue =.017), nursing students who had experience with needle sticks and those who didn't (P-value =.015).

**Conclusions:**

The authors recommend that clinical training departments and universities continue to offer occupational health and safety courses and training for health science students. Since the training had a positive impact on the students' practices.

## Introduction

Occupational Health and Safety (OHS) is an interdisciplinary field of action aimed at promoting and maintaining the highest degree of physical, mental, and social well-being of workers in all occupations; protecting workers from deviation from health due to their working conditions; protecting workers in their jobs from risks arising from factors harmful to health; putting the worker in a professional environment that is compatible with his physiological and psychological capabilities (1). There are 3.5 billion people in the world who are workers; around 140 million are in the health and social sector, including physicians, nurses, and other health workers in diverse disciplines (2, 3).

The healthcare system is a specialized and complex system that includes various health workers and units that interact to deliver healthcare (4). Health care systems in countries such as Palestine suffer from the availability of essential drugs, equipment, and diagnostic tests, and the quality of health care is poor(5–7).

Unsafe working conditions are among the causes of poor quality of care and occupational burnout (8, 9). The main occupational hazards that healthcare providers can face are infections (hepatitis, respiratory infections), Ergonomic hazards(back injury, musculoskeletal disorders), chemical hazards(allergy, toxic drugs), radiation hazards, and Psychosocial hazard (1). The World Health Organization has estimated that 2.1% of all deaths and about 3% of the burden of disease globally can be attributed to occupational hazards (10). Oftentimes, accidents do not happen only because of limited knowledge but because safety procedures become routine and individuals ease their precautions (11). Misunderstanding or lack of proper knowledge is a major difference between an experienced person and a novice and an important factor that may lead to an accident (12).

A healthy and safe workplace is supposed to enhance healthcare quality, patient safety, healthcare providers' retention, and sustainability (1). Safety practices help to save health care providers, visitors, and clients from health risks, it includes the provision of immunization against diseases, proper ways to deal with toxic, chemical materials, and use of personal protective equipment (PPE) such as gowns, gloves, masks, and head cover(13–15). With insufficient safety practice, the risks of infection through exposure to bodily fluids are substantial (16).

Similar to the low and middle-income countries, the Palestinian health care system still complains of a deficiency of human resources, particularly nurses, according to the latest annual report (2021) of the Palestinian Ministry of Health, there are 2.2 nurses per 1000 people(17, 18). Nurses are the heart of any healthcare system, they play a vital role in establishing health interventions and overcoming challenges, as health service providers on the front lines, nurses may face various challenges, such as the risk of infection, lack of preventive supplies, and lack of essential medicines (19).

Before starting clinical training in health institutions for Al-Israa University nursing students, all nursing clinical instructors attended a safety precautions course that included (occupational health and safety rules, wearing of personal protective equipment, precautions for handling contaminated and dangerous materials, and hand washing). After that, the nursing clinical instructors were asked to explain this training program to the nursing students before they entered the health institutions.

There is a dearth of research on nursing knowledge, attitudes, and practices (KAP) regarding occupational health and safety procedures. Therefore, this study aims to assess the knowledge, attitudes, and practice of occupational health and safety among nursing students at Al-Israa University.

## Methods

**Study design and setting**: In this cross-sectional study, a structured online questionnaire was distributed from March to May 2021 to collect information from nursing students at Al-Israa University in Gaza Strip (GS). GS is a narrow band of land with a total 365 km2 surface area, located in the south of Palestine, and lying on the coast of the Mediterranean Sea. GS comprises 5 provinces and about 72% of the population are refugees (20). Al- Israa University is an independent, private, and non-for-profit institution. It includes the college of Intermediate studies and five faculties with over fifteen different majors (21).

**Sampling and sample size**: The study population was nursing students at Al-Israa University, who attended at least one semester of clinical training. Due to the low number of students, we used a census sampling, and the sample size was set equal to the population size (n = 350). The inclusion criteria were as follows: nursing students at Al-Israa University either a diploma or a bachelor's degree; attend at least one semester of clinical training. On the other hand, nursing students who had never attended clinical training were excluded.

**Study instrument**: The questionnaire was developed in Arabic language after reviewing the questionnaires of the appropriate previous studies (22–29). A preliminary questionnaire consists of four main parts:

1-Socio-demographic measured using 9 items: gender, age, education program type, academic level, previous experience needle stick or sharp object injury, place of clinical training, the vaccine against hepatitis, attending safety precautions course, and need more occupational health and safety training.

2- Knowledge measured using 11 items: Knowledge about availability of instructions for occupational health and safety rules, emergency procedures, use of first aid equipment, handling of a fire outbreak, and precautions for handling toxic and dangerous substances.

3- Attitudes measured using 22 items: Attitude towards clinical training area safety, safety while working in a team with colleagues, safety rules, the effectiveness of safety training, and the effectiveness of personal protective equipment in preventing infection.

4- Practice measured using 19 items: Organizing the training area, personal protective equipment, the method of handling contaminated and sharps materials, hand washing, the reporting of needle stick injuries, and medical management.

A 5-points Likert scale was used for response categories. For knowledge and attitudes: (1) Strongly disagree; (2) Disagree; (3) Unsure; (4) Agree; (5) Strongly agree. For practice (1) never; (2) rarely; (3) sometimes; (4) often; (5) always.

**Validation of the questionnaire**: Content and Face validity were examined for the questionnaire independently validated by ten experts (Health experts, Head nurses, researchers, clinical instructors, and academics). The content validity index was calculated to assess the relevance of the questionnaire questions (30). All questions were rated as relevant. Minor changes were made to the language and structure of the questions based on an agreement between the author (AFQ), two head nurses, and a clinical nursing instructor. After that, the draft was piloted among 30 eligible nursing students. The results of the pilot study showed a good overall Cronbach's alphas of .82.

**Data collection**: The link to the study questionnaire was sent to nursing students through the Telegram and WhatsApp training groups. Participating nursing students were asked to forward the link to their other colleagues in the department. To achieve a high response rate, the study questionnaire was sent several times, and the link was available for approximately 3 months.

**Data analysis**: Data were analyzed using the software IBM-SPSS (Statistical Package for Social Sciences), version 22. Negatively worded item codes were reversed before data was entered and analyzed. Descriptive statistics summarized the Characteristics of nursing students. Percentages and frequencies were used to summarize categorical variables, while mean scores and standard deviations (SD) were used to represent continuous variables. Independentsamples T-Test and ANOVA test were used to compare means. All statistical tests were performed at the 0.05 level of statistical significance.

**Ethics approval and consent to participate**: The study was approved by the ethics committee at Al-Israa University. In addition, informed consent was collected from all participants before completing the questionnaire.

## Results

**Characteristics of the study participants**: The characteristics of participating nursing students are summarized in Table 1. Of the 350 eligible students, 219 students answered the online questionnaire, yielding a response rate of 62.6%. The majority of participants were female (81.7%), studying in a diploma program (70.8%). 21% of nursing students claimed that they have experienced a needle stick or sharp object injury. More than 62% of the study participants believed that they urgently need more occupational health and safety training.

**Table 1:** Characteristics of the study participants

Variables	Participants (n=219)	Percentage (%)
**Gender**		
Male	40.0	18.3
Female	179	81.7
**Age**		
18–19 years	58.0	26.5
20–21 years	89.0	46.0
22–24 years	42.0	19.2
> 25 years	30.0	13.7
**Education Program Type**		
Diploma	155	70.8
Bachelor	64.0	29.2
**Academic level**		
First year	74.0	33.8
Second year	82.0	37.4
Third year	28.0	12.8
Fourth year	35.0	16.0
**Previous experience of a needle stick or sharp object injury**		
Yes	46.0	21.0
No	173	79.0
**Place of clinical training**		
Government Hospital(GH)	168	76.7
Primary Health Center(PHC)	16.0	07.3
UNRWA center	06.0	02.7
All of the above	29.0	13.2
**Completed Vaccine Against Hepatitis**		
Yes	73.0	33.8
No	145	66.2
**Attended Safety precautions course during training**		
Yes	197	89.9
No	22.0	10.1
**Need more occupational health and safety training**		
Yes	136	62.1
No	83.0	37.9

**The study participants' knowledge of occupational health and safety**: Table 2 displays that the mean scores of knowledge among nursing students regarding occupational health and safety were M±SD:78.2% ±12.9. The vast majority of participants (93.2%) claimed that they know that there are procedures for emergencies inside the clinical training area, 93.2% know how to use first aid equipment, around 80% know a proper way to when exposed to a burn or contact with a hot object, and around 60% of participants claimed that they know the precautions for handling toxic and dangerous materials.

**Table 2:** The study participants' knowledge of occupational health and safety

Items	5&4 N (%)	3 N (%)	2&1 N (%)
In the training clinical area, there are instructions for occupational health and safety rules	197(89.9)	16(07.3)	06(02.7)
There are procedures for emergencies inside the clinical training area	204(93.2)	10(04.6)	05(02.3)
I know how to use first aid equipment during clinical training	190(86.8)	17(7.8)	12(5.5)
I know what to do when a fire breaks out in the clinical training area	166(75.8)	30(13.7)	23(10.5)
I know what should do if a chemical gets into my eyes	152(69.4)	45(20.5)	22(10.1)
I know how to use a fire blanket in case a fire breaks out in the training area	168(76.8)	26(11.9)	25(11.4)
I know how to use a fire extinguisher in case a fire breaks out in the training area	133(60.7)	41(18.7)	45(20.5)
I know the first aid required when chemicals or drugs are swallowed	146(66.6)	41(18.7)	32(14.6)
I know what to do if chemicals touch my skin	142(64.8)	46(21.0)	31(14.2)
I know how to deal when exposed to a burn or contact with a hot object	175(79.9)	28(12.8)	16(7.3)
I know the precautions for handling toxic and dangerous materials	130(59.4)	58(26.5)	31(14.2)
**M ±SD:78.2 ±12.9**			

Data are expressed as percentages and frequencies. M: Mean scores, SD: Standard Deviation. (1 = strongly disagree, 2 = Disagree, 3 = Unsure, 4 = Agree, 5 = strongly agree).

**Attitudes of the study participants regarding occupational health and safety**: Table 3 displays that the mean scores of attitude among nursing students regarding occupational health and safety was M±SD:80.6% ±7.1. Thevast majority of participants' (98.2%) claimed that they benefit from their past experiences to prevent mistakes, 96.8%claimed that having clear safety rules reduces the rate of accidents, feel safe during work in a team, and 97.2% believed that safety training in the hospital can have a significant impact in preventing accidents. In addition, only 20.5% of participants believed that wearing gloves when taking blood samples is a waste of time.

**Table 3:** Attitudes of the study participants regarding occupational health and safety

Items	5&4 N (%)	3 N (%)	2&1 N (%)
Feel safe when I work with my colleagues in a team	212(96.8)	06(2.7)	01(.5)
Have complete confidence in the ability of my colleagues to deal safely during clinical training	194(88.5)	17(7.8)	08(3.7)
Benefit from my past experiences to prevent mistakes	215(98.2)	03(1.4)	01(.5)
Take my colleagues' opinion and their views on issues related to the dangers of hospitals and training	206(94.1)	11(5.0)	02(1.0)
We talk to each other about safety only when an accident occurs	128(58.4)	47(21.5)	44(20.1)
believe that having clear safety rules reduces the rate of accidents	212(96.8)	06(2.7)	01(.5)
Safety training in the hospital can have a significant impact on preventing accidents	213(97.2)	5.0(2.3)	01(.5)
The periodic evaluation of hospital safety procedures by the administration contributes to discovering future risks	203(92.7)	14(6.4)	02(1.0)
In the hospital, I work hard to achieve the highest levels of occupational health and safety	213(97.2)	5.0(2.3)	01(.5)
In the hospital, I care about the safety of others	173(78.9)	10(4.6)	36(16.4)
In the hospital, I care about treating the dangers that may reveal	167(76.2)	14(6.4)	38(17.4)
In the hospital, I deal with small accidents as if they were a normal part of our work	157(71.7)	30(13.7)	32(14.6)
Can accept dangerous behavior as long as it does not result in accidents	125(57.1)	38(17.3)	56(25.6)
I am safe from being infected by infectious blood diseases	105(47.9)	48(21.9)	66 (30.1)
Wearing gloves when taking blood samples is a waste of time	45(20.5)	25(11.4)	149(68.1)
The university focuses on occupational health and safety rules for students during training	193(88.1)	18(8.2)	08(3.7)
The training area is safe	180(82.2)	27(12.3)	12(5.5)
Use personal protective equipment in case of emergency	127(58.0)	45(20.5)	47(21.5)
The use of gloves while collecting samples from patients is a useful strategy to reduce infections	203(92.6)	08(3.7)	08(3.7)
I understand the procedures for dealing with accidents or injuries that may occur while doing my job as a nurse	201(91.8)	14(6.4)	04(1.8)
The Department of Training adequately trains students on accident prevention in hospitals	194(88.6)	18(8.2)	07(3.2)
The hospital administration makes sure that everyone in the hospital has sufficient information about safety rules	157(71.7)	43(19.6)	19(8.7)
**M±SD:80.6 ±7.1**			

Data are expressed as percentages and frequencies. M: Mean scores, SD: Standard Deviation. (1 = strongly disagree, 2 = Disagree, 3 = Unsure, 4 = Agree, 5 = strongly agree).

**The study participants' practice of occupational health and safety**: Table 4 displays that the mean scores of practice among nursing students regarding occupational health and safety was M±SD:81.2% ±7.6. The vast majority of participants (97.2%) claimed that they keep the training area clean and organized,96.3% wash their hands if they touch blood or secretions of the patient's body with soap and water, 95.4% use a safety box to dispose of needles, scalpels, 94.1% Take off their watch, jewelry, and ring when washing hands and 93.2% claimed that they wear a hospital coat to protect their clothes during the training, and believed that safety training in the hospital could have a significant impact in preventing accidents. In addition, only 20.5% of participants believed wearing gloves when taking blood samples is a waste of time.

**Table 4:** The study participants' practice of occupational health and safety

Items	5&4 N (%)	3 N (%)	2&1 N (%)
Keep the training area clean and organized	213(97.2)	03(1.4)	03(1.4)
Adhere to wearing a medical mask during clinical training	214(97.7)	03(1.4)	02(0.9)
Handle contaminated materials correctly to prevent infection	168(76.7)	13(5.9)	38(17.4)
Wear a hospital coat to protect my clothes during the training	204(93.1)	09(4.1)	06(2.8)
Wash your hands when starting work	178(81.3)	16(7.3)	25(11.4)
Take off your watch, jewelry, and ring when washing your hands	206(94.1)	09(4.1)	04(1.8)
Wash your hands if you touch blood or any fluids or secretions of the patient's body with soap and water	211(96.3)	03(1.4)	05(2.3)
Read the description of the drug when dealing with it	207(94.5)	10(4.6)	02(0.9)
change the gloves when contact with unsterile materials	162(74.0)	14(6.4)	43(19.6)
Wash your hands at end of the training day	180(82.2)	15(6.8)	24(11.0)
Use the safety box to dispose of needles, scalpels	209(95.4)	04(1.8)	06(2.7)
Wear gloves when handling contaminated items or dealing with body secretions	202(92.2)	05(2.3)	12(5.5)
Remove the needle cover before disposal	123(56.2)	32(14.6)	64(29.2)
Recap needles carefully before disposing of it	44(20.1)	24(11.0)	151(68.9)
Separate the needle from the syringe before disposal	133(60.7)	17(7.8)	69(31.5)
Inform the clinical instructor about ta needle stick injury during training	188(85.8)	19(8.7)	12(5.5)
Follow the professional rules for the disposal of medical waste	200(91.3)	12(5.5)	07(3.2)
Wear a head cover before doing any procedure inside the hospital	177(80.1)	26(11.9)	16(7.3)
Wear shoes that cover the foot	159(72.6)	27(12.3)	33(15.1)
**M±SD:81.2 ±7.6**			

Data are expressed as percentages and frequencies. M: Mean scores, SD: Standard Deviation. (1= Never, 2= Rarely, 3= Sometimes, 4= often, 5= Always).

**Characteristics of the study participants in relation to knowledge, attitudes, and practice of occupational health and safety**: Table 5 demonstrated that in terms of knowledge, the mean scores were statistically significant between nursing students who attended a safety precautions course during clinical training and those who didn't attend *P-value =.002.* The nursing students who attended a training course were more knowledgeable with mean scores of 79.3%, SD±12.1.

**Table 5:** Characteristics of the study participants in relation to knowledge, attitude, and practice of occupational health and safety

Variables	Knowledge Mean ± SD	*P-value*	Attitude Mean ± SD	*P-value*	Practice Mean ± SD	*P-value*
**Gender**						
Male	78.4±12.4		81.0±7.8		78.6±8.5	
Female	78.2±13.0	.936	80.5±6.9	.710	81.8±7.3	***.017***
**Age**						
18–19 years	75.5±13.8		80.9±6.6		81.1±6.4	
20–21 years	80.1±12.7		80.8±7.8		81.3±8.8	
22–24 years	79.9±11.0	.082	80.9±7.1	.504	81.2±7.3	.992
> 25 years	75.3±12.9		78.8±5.8		81.2±6.2	
**Education Program Type**						
Diploma	78.1±13.8	.854	81.3±6.9	***.026***	81.1±7.6	.968
Bachelor	78.4±10.4		78.9±7.3		81.2±7.5	
**Academic level**						
First year	77.2±15.6		81.3±7.4		81.5±6.8	
Second year	79.1±11.9		81.3±6.8		81.6±7.9	
Third year	78.7±11.3	.818	77.8±6.8	.055	79.4±7.5	.589
Fourth year	77.8±9.7		79.3±7.4		81.2±8.5	
**Needle stick or sharp object injury**						
Have experience	75.8±11.3		79.7±8.6		81.7±7.1	
Don't have experience	78.6±13.0	.296	80.8±6.7	.433	77.8±10.0	***.015***
**Place of clinical training**						
Government hospital	77.1±13.6		80.4±7.5		81.1±7.3	
Primary Health Center(PHC)	81.7±8.5		79.8±5.8		82.8±7.5	
UNRWA center	80.9±8.2		84.5±8.9		82.6±7.9	
All of the above	82.5±9.9	.203	81.9±5.3	.626	81.5±8.9	.633
***Safety precautions course during clinical training***						
have attended	79.3±12.1	***.002***	80.9±6.8	***.017***	81.6±7.1	***.016***
Did not attend	65.9±14.8		76.7±6.1		77.1±10.9	

SD: standard deviation; UNRWA: United Nations Relief and Works Agency; all of the above: (GH, PHC, and UNRWA center). Data are expressed as mean values ± SD for continuous variables. The differences between means were tested using the independent sample t-test and one-way ANOVA

In terms of attitudes, the mean scores were statistically significant between diploma and bachelor students (P-value =.026), as well as nursing students who attended a safety precautions course during clinical training and those who did not (P-value =.017). Students who are studying diploma program have a more positive attitude compared to bachelor students with mean scores of 81.3%, SD±6.9.

In terms of practice, the mean scores were statistically significant between males and females (P-value =.017), nursing students who had experience with needle sticks or sharp object injury and those who did not (P-value =.015), and nursing students who attended a safety precautions course during clinical training and those who did not (P-value =.016). Females' students more practice safety precautions compared to males (81.8%, SD±7.3). In addition, nursing students who had experience with needle sticks or sharp object injury more practice safety precautions compared to thosewho did not (81.7%, SD±7.1).

## Discussion

Health and safety in the workplace are critical components in healthcare institutions. Unsafe working conditions are among the causes of poor quality of care and occupational burnout among health care providers (8, 9). Nurses frontline health service providers, nurses may face different challenges, such as the risk of infection, lack of protective supplies, and lack of essential medications (19). This study aims to assess the levels of knowledge, attitude, and practice of occupational health and safety among nursing students at Al-Israa University. In the present study that the mean scores of knowledge Attitudes and practice among nursing students were M±SD:78.2% ±12.9, M±SD:80.6% ±7.1, M±SD:81.2% ±7.6 respectively.

In terms of knowledge, attitude, and practice, the mean scores were statistically significant between nursing students who attended a safety precautions course during clinical training and those who didn't attend *P-value <.05.*

In terms of practice, the mean scores were statistically significant between males and females (P-value =.017). In addition, the mean scores were statistically significant between nursing students who had experience needle sticks or sharp object injury and those who didn't (P-value =.015).

A previous study conducted among Palestinian nurses and midwives showed that they have a high level of knowledge and practice of safety precautions (74.6% and 83.8% respectively) (31). In addition, a study conducted among hospital healthcare providers in Cyprus demonstrated that 57.5% of the healthcare providers had a good level of knowledge, 37.3% positive attitude, and 30.9% had good practice toward safety precautions (26). In Nigeria, a study conducted among healthcare providers demonstrated that 81.8%% of the healthcare providers had a good level of knowledge, 56.2% positive attitude, and 70.1%% had a good practice toward safety precautions (32). A possible explanation for the high mean scores among nursing students can be that around 90% of the study participants claimed that they attended a safety precautions course during training, and 88.6%reported that the department of clinical training adequately trains students on accident prevention in hospitals. In addition, 71.7% announced that the hospital administration makes sure that everyone in the hospital has sufficient information about safety rules.

In terms of knowledge, attitude, and practice, the mean scores were statistically significant between nursing students who attended a safety precautions course during clinical training and those who didn't attend *P-value* <*.05.*97.2% believed safety training in the hospital can have a significant impact on preventing accidents. Our results were consistent with previous studies that supported the benefits of education and training, previous systematic reviews demonstrated the positive and significance of training interventions in occupational health and safety on knowledge, attitudes, and behaviors of participants(33, 34). In terms of practice, the mean scores were statistically significant between males and females (P-value =.017), females students more practice safety precautions compared to males. Our results are consistent with studies conducted in Nigeriaand inconsistent with a study conducted among nursing students in Turkey (29, 32, 35).

In addition, the mean scores were statistically significant between nursing students who had experience needle sticks or sharp object injury and those who didn't (P-value =.015). Our results are inconsistent with a study conducted among nurses in china that demonstrated less exposure experience leads to higher practice and compliance (36).

During practical training, students are usually exposed to various risks. The most common exposure was needle stick injury (NSI) with a rate of 21% when syringes and other sharp devices were used. The prevalence of NSI among students of health-related fields in other countries appears to be common. For example, the prevalence of NSI among Pakistani dental students was between 30% and 73%(37). Our results are higher than that of nursing students in South Africa (16%) (38) and lower than nursing and midwives students in Iran (30.1%) (39).

Moreover, 11.4% of the study participants do not wash both hands and 19.6% do not change their gloves when they touch something that is not sterile. The result showed that the habit of hygiene among students during training needs to be organized, and students must be made aware of the importance of this habit to prevent infectious diseases. Lack of enough practice about hand hygiene is one of the major risks of spread of infection in hospitals and the attitude of students toward proper hand hygiene also influences the way they adhere to hand washing and wearing gloves (40)

About 20.1% of respondents reported that they recap needles cover before disposal. About 56.2% of the respondents reported that they removed the needle cover before disposal, and 60.7% of them reported that they separated the needles from the syringes before disposal, which means that they did not follow the protocols to reduce the risk of blood-borne pathogens. Prevention of occupational exposure to healthcare waste includes strict adherence to universal precautions and standard methods for the separation and disposal of healthcare waste (41). Our results are similar to those of another study that showed that 11% of students were unaware that the virus could be transmitted through infected needles and found that 44% of students would destroy the needle with a needle destroyer and 15% would destroy it in a puncture-resistant container with disinfectant (42).

Only 33.8% of study participants reported completing their hepatitis vaccinations. A previous study conducted among healthcare providers in India showed that 36% completed hepatitis B vaccination (43). In Saudi Arabia, 91.4% of dental students reported receiving a vaccine against hepatitis (44). Deans of nursing colleges, in collaboration with the heads of clinical training departments, should ensure that nursing students complete the hepatitis vaccination before commencing training in health facilities.

## Strengths and Limitations of the study

A possible limitation of the current study is only nursing students with access to the Internet could contribute to this study. Also, other healthcare professionals, such as medical students, lab- technicians were not included in this study. In addition, this study is a cross-sectional study design that could limit the generalizability of study results. Finally, the study is based on selfreported data that can lead to recall bias and social desirability bias. Despite these limitations, this study gave preliminary results about KAP towards occupational health and safety among frontline health service providers, these results can assist policy and decision-makers in the design and development of appropriate occupational health and safety programs.

In conclusion, the authors recommend that clinical training departments and universities continue to offer occupational health and safety courses and training for health science students. Since the training had a positive impact on the students' practices. Nursing academics and educators are advised to adjust the curriculum according to the new educational needs of nursing by adding principles of occupational health and safety precautions. Further nationwide studies on the OHS topic among various students of health colleges and health care institutions should be conducted

## References

[1] WHO (2022). Caring for those who care: guide for the development and implementation of occupational health and safety programmes for health workers: executive summary.

[2] Organization IL (2020). ILO Monitor: COVID-19 and the World of Work.

[3] Dobbs R, Madgavkar A, Barton D, Labaye E, Manyika J, Roxburgh C (2012). The world at work: Jobs, pay, and skills for 3.5 billion people.

[4] Johnson JK, Miller SH, Horowitz SD (2008). Systems-based practice: improving the safety and quality of patient care by recognizing and improving the systems in which we work. Advances in patient safety: new directions and alternative approaches (Vol 2: culture and Redesign).

[5] Albelbeisi AH, Albelbeisi A, El Bilbeisi AH, Taleb M, Takian A, Akbari-Sari A (2021). Public Sector Capacity to Prevent and Control of Noncommunicable Diseases in Twelve Lowand Middle-Income Countries Based on WHO-PEN Standards: A Systematic Review. Health Services Insights.

[6] Leslie HH, Spiegelman D, Zhou X, Kruk ME (2017). Service readiness of health facilities in Bangladesh, Haiti, Kenya, Malawi, Namibia, Nepal, Rwanda, Senegal, Uganda and the United Republic of Tanzania. Bulletin of the World Health Organization.

[7] Albelbeisi AH, Albelbeisi A, El Bilbeisi AH, Takian A, Akbari-Sari A (2020). Capacity of Palestinian primary health care system to prevent and control of non-communicable diseases in Gaza Strip, Palestine: A capacity assessment analysis based on adapted WHO-PEN tool. The International Journal of Health Planning and Management.

[8] Russo G, Xu L, McIsaac M, Matsika-Claquin MD, Dhillon I, McPake B (2019). Health workers' strikes in low-income countries: the available evidence. Bulletin of the World Health Organization.

[9] Hall LH, Johnson J, Watt I, Tsipa A, O'Connor DB (2016). Healthcare staff wellbeing, burnout, and patient safety: a systematic review. PloS one.

[10] WHO (2018). Preventing disease through a healthier and safer workspace September.

[11] Karapantsios T, Boutskou E, Touliopoulou E, Mavros P (2008). Evaluation of chemical laboratory safety based on student comprehension of chemicals labelling. Education for chemical engineers.

[12] Wiediger SD, Hutchinson JS (2002). The significance of accurate student self-assessment in understanding of chemical concepts. Journal of chemical education.

[13] Valim MD, Marziale MHP, Richart-Martínez M, Sanjuan-Quiles Á (2014). Instruments for evaluating compliance with infection control practices and factors that affect it: an integrative review. Journal of Clinical Nursing.

[14] Goswami HM, Soni ST, Patel SM, Patel MK (2011). A study on knowledge, attitude and practice of laboratory safety measures among paramedical staff of laboratory services. National Journal of Community Medicine.

[15] Vaz K, McGrowder D, Alexander-Lindo R, Gordon L, Brown P, Irving R (2010). Knowledge, awareness and compliance with universal precautions among health care workers at the University Hospital of the West Indies, Jamaica. International Journal of Occupational & Environmental Medicine.

[16] Ababa A (2012). Infection prevention and patient safety reference manual for service providers and managers in healthcare facilities of Ethiopia.

[17] Ministry of Heath (2021). Annual Health Report for Southern Governorates. Gaza strip, Palestine.

[18] Albelbeisi AH, Albelbeisi A, El Bilbeisi AH, Taleb M, Takian A, Akbari-Sari A (2021). Barriers of adherence among Palestinian healthcare professionals towards the protocol of health education and counselling on healthy behaviours for non-communicable diseases. Ethiopian Journal of Health Sciences.

[19] Courbage Y, Abu Hamad B, Zagha A (2016). Palestine 2030-demographic change: opportunities for development.

[20] PCBS (2017). Paletine in Figures 2016.

[21] ISRAA UNIVERSITY Israa Profile [01-06-2022].

[22] Ngah H, Mohd Hairon S, Hamzah NA, Noordin S, Shafei MN (2022). Development and Validation of Knowledge, Attitude, and Practice Questionnaire: Toward Safe Working in Confined Spaces. International Journal of Environmental Research and Public Health.

[23] Yasmeen MS, Ali TS, Khalid W, Kurji MZ, Hazara MSM, Bashir MS (2022). Knowledge and Practices Regarding Standard Precautions for Infection Control Among Nurses Working at a Public, Tertiary Care Hospital Islamabad, Pakistan. National Journal of Community Medicine.

[24] Rezaei M-S, Golbabaei F, Behzadi MH (2017). Assessing the healthcare workers' knowledge, attitude, and practice toward health, safety, and environment in an educational hospital affiliated by Iran university of medical sciences (2012–2013). Journal of Environmental Science and Technology.

[25] Sabbah I, Sabbah H, Sabbah S, Akoum H, Droubi N (2013). Occupational exposures to blood and body fluids (BBF): assessment of knowledge, attitude and practice among health care workers in general hospitals in Lebanon.

[26] Abuduxike G, Vaizoglu SA, Asut O, Cali S (2021). An assessment of the knowledge, attitude, and practice toward standard precautions among health workers from a hospital in northern cyprus. Safety and health at work.

[27] Awan A, Afzal M, Majeed I, Waqas A, Gilani SA (2017). Assessment of knowledge, attitude and practices regarding occupational hazards among Nurses at Nawaz Sharif Social Security Hospital Lahore Pakistan. Saudi Journal of Medical and Pharmaceutical Scinces.

[28] Abd Elaziz K (2009). Assessment of knowledge, attitude and practice of hand washing among health care workers in Ain Shams University. J Prev Med Hyg.

[29] Eyi S, Eyi İ (2020). Nursing students' occupational health and safety problems in surgical clinical practice. SAGE Open.

[30] Lynn MR (1986). Determination and quantification of content validity. Nursing research.

[31] Fashafsheh I, Ayed A, Koni M, Hussein S, Thultheen I (2016). Midwives and nurses compliance with standard precautions in Palestinian hospitals. Open Journal of Nursing.

[32] Adebimpe WO (2016). Knowledge, attitude, and practice of use of safety precautions among health care workers in a Nigerian tertiary hospital, 1 Year after the Ebola virus disease epidemic. Annals of global health.

[33] Robson LS, Stephenson CM, Schulte PA, Amick III BC, Irvin EL, Eggerth DE (2012). A systematic review of the effectiveness of occupational health and safety training. Scandinavian journal of work, environment & health.

[34] Ricci F, Chiesi A, Bisio C, Panari C, Pelosi A (2016). Effectiveness of occupational health and safety training: A systematic review with meta-analysis. Journal of Workplace Learning.

[35] Aluko OO, Adebayo AE, Adebisi TF, Ewegbemi MK, Abidoye AT, Popoola BF (2016). Knowledge, attitudes and perceptions of occupational hazards and safety practices in Nigerian healthcare workers. BMC research notes.

[36] Luo Y, He G-P, Zhou J-W, Luo Y (2010). Factors impacting compliance with standard precautions in nursing, China. International Journal of Infectious Diseases.

[37] Pervaiz M, Gilbert R, Ali N (2018). The prevalence and underreporting of needlestick injuries among dental healthcare workers in Pakistan: A systematic review. International journal of dentistry.

[38] Zungu LI, Sengane M, Setswe KG (2008). Knowledge and experiences of needle prick injuries (NPI) among nursing students at a. South African Family Practice.

[39] Khoshnood Z, Nouhi E, Mahdi SA (2015). Prevalence of needle stick and sharp injuries among nursing and midwifery students. Asian Journal of Nursing Education and Research.

[40] Sebong PH, Tjitradinata C, Goldman RE (2021). Promoting COVID-19 prevention strategies in student dormitory setting: A qualitative study. Journal of American College Health.

[41] Polan MAA, Al Noman N, Jan CM, Hasan MR, Saito T (2013). Practice and knowledge of health personnel on impact of medical wastes in Upazilla Health Complexes under Dhaka Division in Bangladesh. City Dental College Journal.

[42] Guruprasad Y, Chauhan DS (2011). Knowledge, attitude and practice regarding risk of HIV infection through accidental needlestick injuries among dental students of Raichur, India. National journal of maxillofacial surgery.

[43] Gupta P, Rakshit P, Gupta RK, Bhatt N, Dutta R, Sherwal B (2017). Assessment of knowledge, attitude, and practices towards occupational injuries infections of healthcare workers at tertiary care hospita. lInternational Journal of Medical Research & Health Sciences.

[44] Al-Shamiri H-M, AlShalawi F-E, AlJumah TM, AlHarthi M-M, AlAli E-M, AlHarthi H-M (2018). Knowledge, attitude and practice of hepatitis B virus infection among dental students and interns in Saudi Arabia. Journal of clinical and experimental dentistry.

